# Supplementary material for: A retrospective analysis of the peer review of more than 75,000 Marie Curie proposals between 2007 and 2018
Source: eLife. 2021 Jan 13;10:e59338. doi: 10.7554/eLife.59338 (PMC7806263; doi:10.7554/eLife.59338)
Supplement: Supplementary file 2. [file elife-59338-supp2.docx]

**Supplementary file 2 for**

Pina DG, Buljan I, Hren D, Marušić A. 2021. Meta-Research: A retrospective analysis of the peer review of more than 75,000 Marie Curie proposals between 2007 and 2018. *eLife* **10**:e59338

**Table A1. Distribution of Marie Curie proposals and their Pearson’s correlation coefficients (*r*_CR/AVIER_), mean difference between CR and AVIER scores, and mean AD indices, by type of actions and by call year**

| **Type of action, call year and panels** | ***r*_CR/AVIER_*** | **Mean Difference**  **CR-AVIER (SD)** | **Mean AD index (SD)** |
| --- | --- | --- | --- |
| **IF – all years** | **0.937** | **-0.02 (4.32)** | **6.96 (4.56)** |
| 2007 (n=1686) | 0.964 | 0.47 (2.66) | 6.13 (4.00) |
| 2008 (n=1753) | 0.948 | 0.68 (2.78) | 5.87 (3.83) |
| 2009 (n=2392) | 0.962 | 0.15 (3.01) | 6.21 (3.99) |
| 2010 (n=2835) | 0.954 | 0.47 (3.14) | 6.13 (4.05) |
| 2011 (n=3302) | 0.951 | 0.38 (3.19) | 6.25 (4.17) |
| 2012 (n=3708) | 0.957 | 0.22 (2.97) | 5.90 (3.77) |
| 2013 (n=4917) | 0.961 | 0.41 (2.99) | 5.99 (3.89) |
| 2014 (n=7397) | 0.935 | 0.03(4.40) | 6.96 (4.51) |
| 2015 (n=8364) | 0.929 | 0.14 (4.77) | 7.13 (4.60) |
| 2016 (n=8805) | 0.929 | -0.08 (4.74) | 7.49 (4.80) |
| 2017 (n=8940) | 0.933 | -0.34 (4.87) | 7.70 (4.93) |
| 2018 (n=9658) | 0.930 | -0.43 (5.12) | 7.59 (4.86) |
| **ITN – all years** | **0.920** | **-0.05 (4.57)** | **7.05 (4.41)** |
| 2008 (n=886) | 0.952 | 0.14 (3.57) | 7.39 (4.50) |
| 2010 (n=858) | 0.949 | 0.01 (4.12) | 7.06 (4.11) |
| 2011 (n=909) | 0.949 | 0.10 (3.94) | 7.12 (4.60) |
| 2014 (n=1149) | 0.931 | -0.06 (4.29) | 7.13 (4.28) |
| 2015 (n=1558) | 0.903 | 0.21 (4.94) | 7.06 (4.58) |
| 2017 (n=1702) | 0.895 | -0.56 (4.84) | 6.79 (4.33) |
| 2018 (n=1634) | 0.871 | -0.04 (5.16) | 6.91 (4.23) |
| **IAPP/RISE – all years** | **0.930** | **-0.05 (4.57)** | **8.51 (5.00)** |
| 2007 (n=102) | 0.978 | -0.35 (3.54) | 8.21 (5.16) |
| 2008 (n=141) | 0.979 | -0.73 (3.04) | 7.54 (4.12) |
| 2009 (n=356) | 0.972 | -0.07 (3.61) | 8.26 (4.77) |
| 2011 (n=160) | 0.956 | -1.74 (4.53) | 8.31 (4.49) |
| 2014 (n=200) | 0.921 | -2.56 (6.40) | 9.83 (5.54) |
| 2015 (n=361) | 0.912 | -1.52 (6.34) | 8.77 (5.25) |
| 2016 (n=366) | 0.909 | 1.14 (6.42) | 8.71 (5.19) |
| 2017(n=321) | 0.918 | -1.82 (6.17) | 8.48 (5.25) |
| 2018 (n=272) | 0.904 | -2.50 (6.63) | 8.02 (4.46) |

SD – standard deviation

*P<0.001 for all.

**Table A2. Distribution of Marie Curie proposals with large CR-AVIER absolute difference, and their mean CR scores, mean CR-AVIER difference and mean AD indices, by type of action and by call year**

|  | **Mean CR score (M, SD)** | **Mean Difference**  **CR-AVIER (SD)** | **Mean AD index (SD)** |
| --- | --- | --- | --- |
| **Overall (n=3,097)** | **67.8 (18.37)** | **-3.40 (13.75)** | **12.86 (6.33)** |
| **All IEF/IF (n=2,482)** | 68.18 (18.60) | -2.84 (13.82) | 13.01 (6.32) |
| **2007 (n=17)** | 66.49 (16.98) | -2.12 (11.76) | 13.94 (6.11) |
| **2008 (n=21)** | 74.95 (16.14) | 8.39 (11.20) | 17.65 (7.86) |
| **2009 (n=27)** | 81.61 (13.51) | 6.62 (10.89) | 14.65 (4.89) |
| **2010 (n=37)** | 75.20 (20.99) | 5.41 (14.34) | 14.89 (8.70) |
| **2011 (n=38)** | 69.72 (19.04) | 2.60 (16.77) | 18.69 (10.08) |
| **2012 (n=41)** | 71.56 (19.28) | 2.67 (12.87) | 14.22 (7.53) |
| **2013 (n=55)** | 66.87 (23.01) | 13.90 (10.07) | 16.25 (6.38) |
| 2014 (n=309) | 67.38 (18.04) | -4.05 (13.55) | 13.08 (5.92) |
| 2015 (n=432) | 70.46 (19.16) | -1.16 (13.95) | 12.62 (6.00) |
| 2016 (n=450) | 70.02 (18.00) | -2.31 (14.06) | 12.89 (5.99) |
| 2017 (n=498) | 65.67 (18.15) | -4.77 (12.96) | 12.73 (6.53) |
| 2018 (n=557) | 66.06 (18.27) | -4.72 (13.77) | 12.41 (5.90) |
| **All ITN (n=417)** | 69.86 (16.79) | -3.69 (13.50) | 12.31 (6.50) |
| **2008 (n=18)** | 71.90(17.40) | 2.42 (13.50) | 16.55 (8.02) |
| **2010 (n=25)** | 61.74 (19.07) | -6.91 (13.69) | 13.24 (4.99) |
| **2011 (n=27)** | 65.04 (17.74) | -5.05 (12.40) | 14.06 (7.29) |
| **2012 (n=34)** | 65.88 (18.56) | -6.56 (13.23) | 14.39 (7.07) |
| 2014 (37) | 69.17 (17.45) | -5.49 (11.96) | 11.67 (5.56) |
| 2015 (83) | 72.00 (17.07) | -1.19 (14.86) | 12.12 (6.39) |
| 2017 (92) | 69.78 (14.62) | -5.88 (12.78) | 11.36 (6.58) |
| 2018 (101) | 73.08 (15.84) | -2.06 (13.32) | 11.42 (6.07) |
| **All IAPP/RISE (n=198)** | 58.28 (16.30) | -9.70 (11.70) | 12.11 (5.88) |
| **2007-2011* (n=23)** | 57.90 (18.92) | -4.69 (11.81) | 13.77 (6.86) |
| 2014 (26) | 58.25 (9.83) | -14.92 (4.84) | 12.72 (5.48) |
| 2015 (39) | 61.21 (15.68) | -9.96 (12.68) | 11.41 (5.32) |
| 2016 (39) | 58.99 (19.37) | -8.02 (13.60) | 12.67 (6.00) |
| 2017 (37) | 60.39 (15.94) | -8.00 (11.92) | 12.48 (6.54) |
| 2018 (34) | 57.92 (16.46) | -12.58 (9.91) | 10.28 (4.95) |

M – mean, SD – standard deviation

*There were only 2 cases in IAPP 2007, 3 cases in 2008, 9 in 2009 and 9 in 2011, so those were merged together due to the sample size.
