# Supplementary material for: A retrospective analysis of the peer review of more than 75,000 Marie Curie proposals between 2007 and 2018
Source: eLife. 2021 Jan 13;10:e59338. doi: 10.7554/eLife.59338 (PMC7806263; doi:10.7554/eLife.59338)
Supplement: Transparent reporting form [file elife-59338-transrepform.pdf]

eLIFE
Westbrook Centre, Milton Road
Cambridge CB4 1YG
UK
P 01223 855340
W elifesciences.org
T @elife

eLife's transparent reporting form

We encourage authors to provide detailed information *within their submission* to facilitate the interpretation and replication of experiments. Authors can upload supporting documentation to indicate the use of appropriate reporting guidelines for health-related research (see EQUATOR Network), life science research (see the BioSharing Information Resource), or the ARRIVE guidelines for reporting work involving animal research. Where applicable, authors should refer to any relevant reporting standards documents in this form.

If you have any questions, please consult our Journal Policies and/or contact us: editorial@elifesciences.org.

Sample-size estimation

- You should state whether an appropriate sample size was computed when the study was being designed
- You should state the statistical method of sample size computation and any required assumptions
- If no explicit power analysis was used, you should describe how you decided what sample (replicate) size (number) to use

Please outline where this information can be found within the submission (e.g., sections or figure legends), or explain why this information doesn't apply to your submission:

We did not perform sample size estimation. All available proposals from 2007 to 2018 were included in analysis (entire population). Described in the methods section.

Replicates

- You should report how often each experiment was performed
- You should include a definition of biological versus technical replication
- The data obtained should be provided and sufficient information should be provided to indicate the number of independent biological and/or technical replicates
- If you encountered any outliers, you should describe how these were handled
- Criteria for exclusion/inclusion of data should be clearly stated
- High-throughput sequence data should be uploaded before submission, with a private link for reviewers provided (these are available from both GEO and ArrayExpress)

Please outline where this information can be found within the submission (e.g., sections or figure legends), or explain why this information doesn't apply to your submission:

All the data analyzed is available in the dataset provided as supplementary data. For H2020, 52 289 proposals were analysed (as explained in the article). Reviewers can perform replications if needed.

Statistical reporting

- Statistical analysis methods should be described and justified
- Raw data should be presented in figures whenever informative to do so (typically when N per group is less than 10)
- For each experiment, you should identify the statistical tests used, exact values of N, definitions of center, methods of multiple test correction, and dispersion and precision measures (e.g., mean, median, SD, SEM, confidence intervals; and, for the major substantive results, a measure of effect size (e.g., Pearson's r, Cohen's d)
- Report exact p-values wherever possible alongside the summary statistics and 95% confidence intervals. These should be reported for all key questions and not only when the p-value is less than 0.05.

Please outline where this information can be found within the submission (e.g., sections or figure legends), or explain why this information doesn't apply to your submission:

For each proposal, we used the Average Deviation (AD) index as a measure of the inter-rater (i.e. inter-reviewer) agreement (Burke, 1999; Burke and Dunlap, 2002) calculated as the average absolute difference between scores of individual reviewers (raters) and the average score of all reviewers. AD indices have the advantage of not requiring the specification of null distribution and gives a value of the inter-rater (dis)agreement in the units of the original scale (0 to 100 in our case), making its interpretation easier and more pragmatic (Smith-Crowe et al., 2013). Therefore, the closer the AD index is to zero, the more agreement there is between the individual reviewers.

Categorical data are presented as aggregated sums, frequencies and percentages, while continuous data as means and standard deviations (for normally distributed data) or medians and interquartile ranges (for non-normally distributed data). The differences between agreement groups were tested with one-way ANOVA, with eta squared as an indicator of the effect size.

The differences between data from the FP7 (2007-2013) and H2020 (2014-2018) were expressed as mean difference and 95% confidence interval (CI). We assessed the associations in each specific criterion between reviewers by using Pearson's correlation coefficient. We compared the CR scores and AD indices from 2007 to 2018 using the interrupted time series analysis and regression analysis using guidance from Cochrane Handbook for the proposals that switched from on-site to remote individual evaluation (ITN and IF actions).

All analyses were done using JASP statistical software (v. 0.11.1.0., JASP Team, 2019), R v.3.6.3. (R Core Team, 2013) and SPSS Statistics for Windows v.19.0 (Armonk, NY: IBM Corp., 2010). Details provided under the Data analysis section of the Methods.

(For large datasets, or papers with a very large number of statistical tests, you may upload a single table file with tests, Ns, etc., with reference to sections in the manuscript.)

Group allocation

- Indicate how samples were allocated into experimental groups (in the case of clinical studies, please specify allocation to treatment method); if randomization was used, please also state if restricted randomization was applied
- Indicate if masking was used during group allocation, data collection and/or data analysis

Please outline where this information can be found within the submission (e.g., sections or figure legends), or explain why this information doesn't apply to your submission:

Groups were created based on the agreements between reviewers.<br><br>In order to have a better understanding on how the level of (dis)agreement between reviewers related to the final CR score, we grouped H2020 proposals based on their IER scores into three groups: i) Proposals with full agreement, where the absolute differences between each of the three pairs of reviewers (i.e. IER1-IER2, IER1-IER3, and IER2-IER3) were equal or below 10 points; ii) Proposals with no agreement, where the absolute differences between each of the three pairs of reviewers were above 10 points; iii) The other proposals, with the cases not included in any of the previous two groups (for example, when one pair of reviewers' IER differ less than or equal to 10 points, and the other two differ more than 10 points, or when two pairs differ less than or equal to 10 points, and the third differ more than 10 points).<br><br>For that analysis based on the scores given by three reviewers, the data related to the ITN call of 2016 were excluded (n=1562) as in that year four reviewers were systematically allocated to evaluate each proposal.

Additional data files ("source data")

- We encourage you to upload relevant additional data files, such as numerical data that are represented as a graph in a figure, or as a summary table
- Where provided, these should be in the most useful format, and they can be uploaded as "Source data" files linked to a main figure or table
- Include model definition files including the full list of parameters used
- Include code used for data analysis (e.g., R, MatLab)
- Avoid stating that data files are "available upon request"

Please indicate the figures or tables for which source data files have been provided:

Dataset is provided as the Supplementary file.
